# Supplementary material for: Multi‐dataset identification of innovative feature genes and molecular mechanisms in keratoconus
Source: J Cell Mol Med. 2024 Sep 19;28(18):e70079. doi: 10.1111/jcmm.70079 (PMC11412914; doi:10.1111/jcmm.70079)
Supplement: Supplementary file 2 — Material S2. [file JCMM-28-e70079-s002.zip › 7.GSEA analysis/2.ATOH7/KEGG_GLYCOLYSIS_GLUCONEOGENESIS.html]

Details for gene set KEGG\_GLYCOLYSIS\_GLUCONEOGENESIS[GSEA]

|  || Dataset | ATOH7.group.cls#h\_versus\_l |
| Phenotype | group.cls#h\_versus\_l |
| Upregulated in class | h |
| GeneSet | KEGG\_GLYCOLYSIS\_GLUCONEOGENESIS |
| Enrichment Score (ES) | 0.43388402 |
| Normalized Enrichment Score (NES) | 1.5869758 |
| Nominal p-value | 0.022821577 |
| FDR q-value | 0.3383528 |
| FWER p-Value | 0.627 |
Table: GSEA Results Summary

  

Fig 1: Enrichment plot: KEGG\_GLYCOLYSIS\_GLUCONEOGENESIS      
 Profile of the Running ES Score & Positions of GeneSet Members on the Rank Ordered List

  

| SYMBOL | TITLE | RANK IN GENE LIST | RANK METRIC SCORE | RUNNING ES | CORE ENRICHMENT || 1 | ALDH2 | na | 221 | 0.659 | 0.0782 | Yes |
| 2 | ADH1C | na | 992 | 0.448 | 0.1136 | Yes |
| 3 | ALDOB | na | 1720 | 0.367 | 0.1397 | Yes |
| 4 | GCK | na | 1752 | 0.364 | 0.1855 | Yes |
| 5 | ALDH1A3 | na | 1890 | 0.353 | 0.2268 | Yes |
| 6 | PGK2 | na | 1991 | 0.345 | 0.2681 | Yes |
| 7 | FBP2 | na | 2527 | 0.307 | 0.2921 | Yes |
| 8 | PCK2 | na | 2919 | 0.285 | 0.3173 | Yes |
| 9 | ENO2 | na | 3413 | 0.259 | 0.3364 | Yes |
| 10 | ADH1A | na | 3425 | 0.259 | 0.3693 | Yes |
| 11 | PGAM2 | na | 4310 | 0.225 | 0.3727 | Yes |
| 12 | GALM | na | 5604 | 0.182 | 0.3588 | Yes |
| 13 | HK2 | na | 5782 | 0.177 | 0.3764 | Yes |
| 14 | LDHAL6B | na | 5965 | 0.172 | 0.3932 | Yes |
| 15 | PCK1 | na | 6929 | 0.145 | 0.3839 | Yes |
| 16 | ALDH9A1 | na | 7741 | 0.125 | 0.3765 | Yes |
| 17 | ALDH3B2 | na | 7831 | 0.122 | 0.3896 | Yes |
| 18 | ALDH1B1 | na | 7850 | 0.122 | 0.4048 | Yes |
| 19 | ENO3 | na | 8533 | 0.108 | 0.3989 | Yes |
| 20 | G6PC2 | na | 8689 | 0.105 | 0.4078 | Yes |
| 21 | PGM1 | na | 9190 | 0.096 | 0.4057 | Yes |
| 22 | LDHA | na | 9500 | 0.090 | 0.4084 | Yes |
| 23 | ADH5 | na | 9514 | 0.090 | 0.4195 | Yes |
| 24 | GPI | na | 10220 | 0.078 | 0.4092 | Yes |
| 25 | ALDH3A1 | na | 10357 | 0.076 | 0.4150 | Yes |
| 26 | LDHC | na | 10530 | 0.073 | 0.4194 | Yes |
| 27 | PKM | na | 10652 | 0.071 | 0.4251 | Yes |
| 28 | PGK1 | na | 11029 | 0.066 | 0.4227 | Yes |
| 29 | HK1 | na | 11153 | 0.064 | 0.4273 | Yes |
| 30 | PFKM | na | 11206 | 0.063 | 0.4339 | Yes |
| 31 | LDHB | na | 11651 | 0.057 | 0.4284 | No |
| 32 | PGAM1 | na | 12162 | 0.051 | 0.4202 | No |
| 33 | ALDOA | na | 12691 | 0.044 | 0.4105 | No |
| 34 | HK3 | na | 13130 | 0.039 | 0.4029 | No |
| 35 | DLAT | na | 13486 | 0.035 | 0.3972 | No |
| 36 | TPI1 | na | 14080 | 0.030 | 0.3839 | No |
| 37 | ALDH7A1 | na | 14436 | 0.026 | 0.3771 | No |
| 38 | GAPDH | na | 14852 | 0.022 | 0.3680 | No |
| 39 | PGAM4 | na | 15239 | 0.019 | 0.3593 | No |
| 40 | PFKL | na | 15515 | 0.017 | 0.3535 | No |
| 41 | BPGM | na | 15857 | 0.014 | 0.3455 | No |
| 42 | PDHA2 | na | 17634 | -0.001 | 0.2943 | No |
| 43 | PDHB | na | 17788 | -0.002 | 0.2902 | No |
| 44 | ADH7 | na | 18056 | -0.005 | 0.2831 | No |
| 45 | PGM2 | na | 18277 | -0.006 | 0.2776 | No |
| 46 | PFKP | na | 18830 | -0.011 | 0.2631 | No |
| 47 | ENO1 | na | 18960 | -0.013 | 0.2610 | No |
| 48 | ACSS2 | na | 19454 | -0.017 | 0.2489 | No |
| 49 | ALDH3A2 | na | 20819 | -0.030 | 0.2134 | No |
| 50 | PDHA1 | na | 21078 | -0.032 | 0.2101 | No |
| 51 | ALDH3B1 | na | 22158 | -0.045 | 0.1847 | No |
| 52 | ACSS1 | na | 24029 | -0.070 | 0.1398 | No |
| 53 | AKR1A1 | na | 24237 | -0.073 | 0.1432 | No |
| 54 | ADH1B | na | 25095 | -0.087 | 0.1296 | No |
| 55 | FBP1 | na | 25972 | -0.103 | 0.1176 | No |
| 56 | ALDOC | na | 26100 | -0.106 | 0.1275 | No |
| 57 | DLD | na | 26919 | -0.123 | 0.1198 | No |
| 58 | LDHAL6A | na | 29599 | -0.198 | 0.0679 | No |
| 59 | ADH4 | na | 29617 | -0.198 | 0.0928 | No |
| 60 | ADH6 | na | 29876 | -0.207 | 0.1119 | No |
| 61 | PKLR | na | 30222 | -0.219 | 0.1300 | No |
Table: GSEA details [plain text format]

  

Fig 2: KEGG\_GLYCOLYSIS\_GLUCONEOGENESIS      
 Blue-Pink O' Gram in the Space of the Analyzed GeneSet

  

Fig 3: KEGG\_GLYCOLYSIS\_GLUCONEOGENESIS: Random ES distribution      
 Gene set null distribution of ES for **KEGG\_GLYCOLYSIS\_GLUCONEOGENESIS**
